# Supplementary material for: Cepharanthine analogs mining and genomes of Stephania accelerate anti-coronavirus drug discovery
Source: Nat Commun. 2024 Feb 20;15:1537. doi: 10.1038/s41467-024-45690-5 (PMC10879537; doi:10.1038/s41467-024-45690-5)
Supplement: Supplementary file 1 — Supplementary Information [file 41467_2024_45690_MOESM1_ESM.pdf]

Cepharanthine analogs mining and genomes of *Stephania* accelerate anti-coronavirus drug discovery

Leng *et al.*

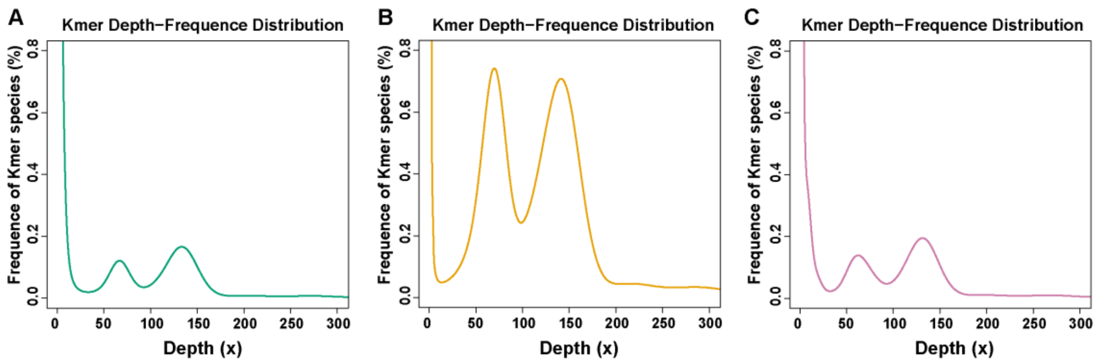

Supplementary Fig. 1. Genome size estimation by *k*-mer analysis.

Genome size estimation of *S. japonica* (A), *S. yunnanensis* (B), and *S. cepharantha* (C) by *k*-mer analysis.

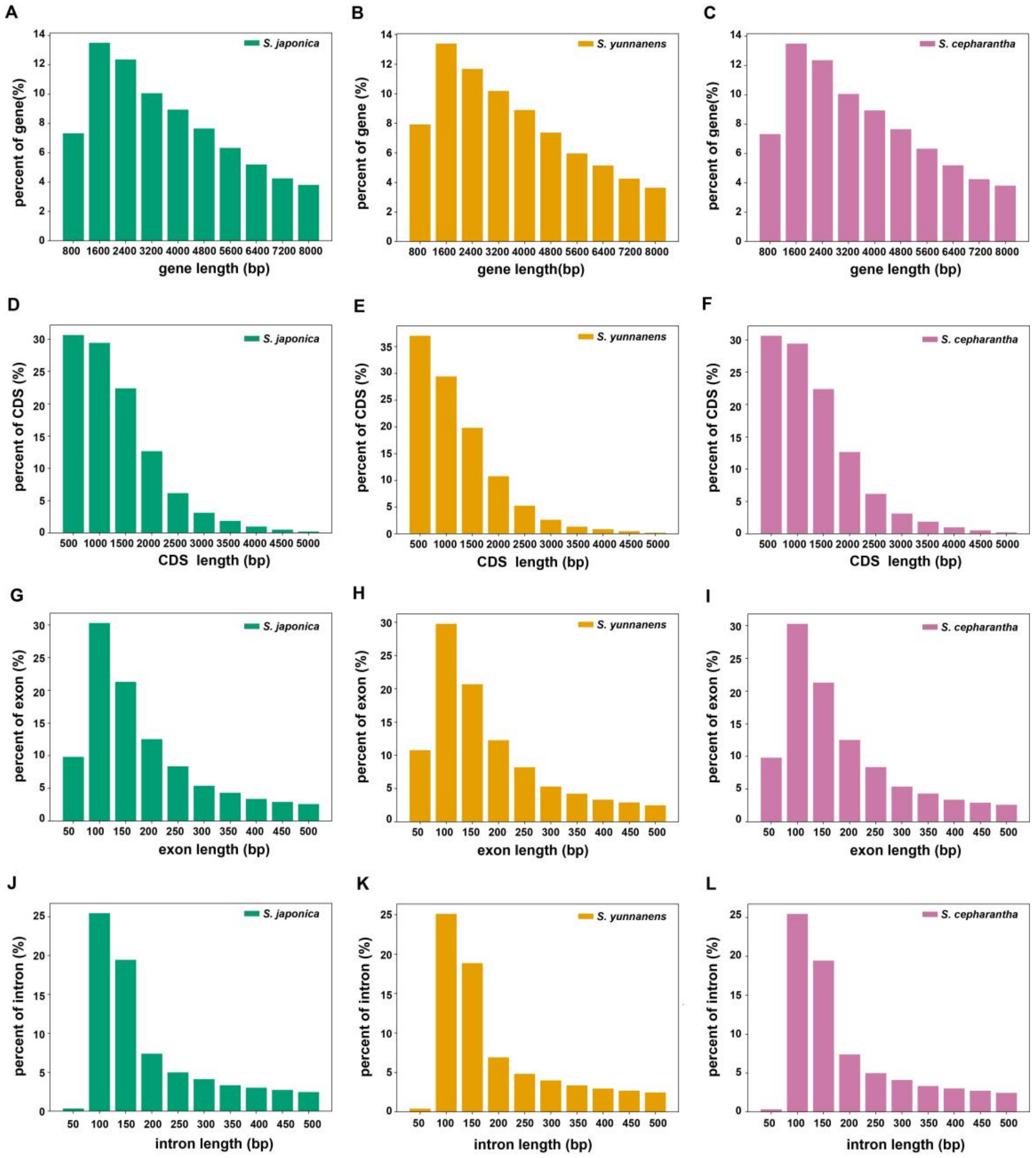

Supplementary Fig. 2. Characteristics of predicted *S. japonica*, *S. yunnanensis*, and *S. cepharantha* protein-coding genes.

A-C, protein coding gene length distribution of *S. japonica*, *S. yunnanensis*, and *S. cepharantha*; D-F, CDS length distribution of *S. japonica*, *S. yunnanensis*, and *S. cepharantha*; G-I, exon length distribution of *S. japonica*, *S. yunnanensis*, and *S. cepharantha*; J-L, intron length distribution of *S. japonica*, *S. yunnanensis*, and *S. cepharantha*. Source data are provided as a Source Data file.

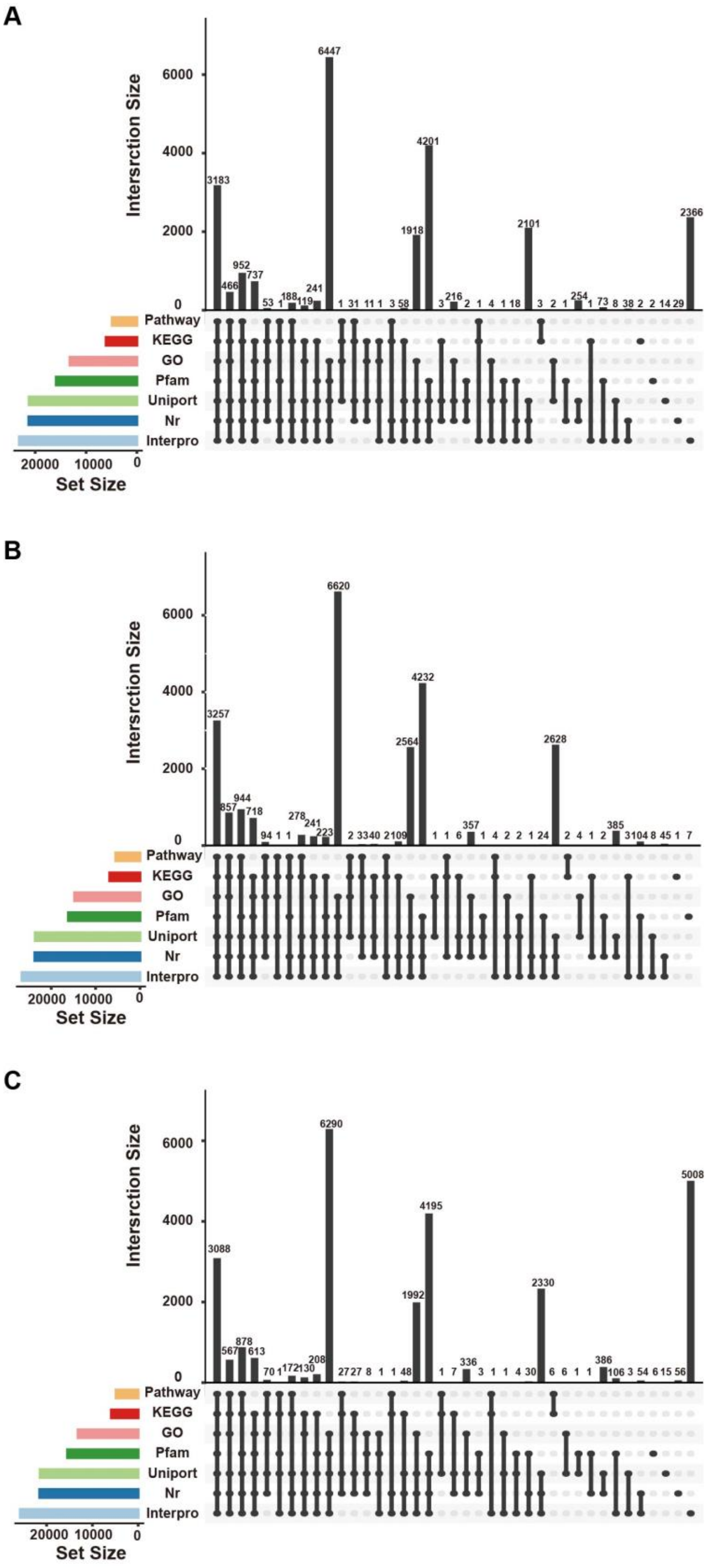

Supplementary Fig. 3. Venn diagram of functional annotation.

Venn diagram of functional annotation of (A) *S. japonica*; (B) *S. yunnanensis*; (C) *S. cepharantha*. Source data are provided as a Source Data file.

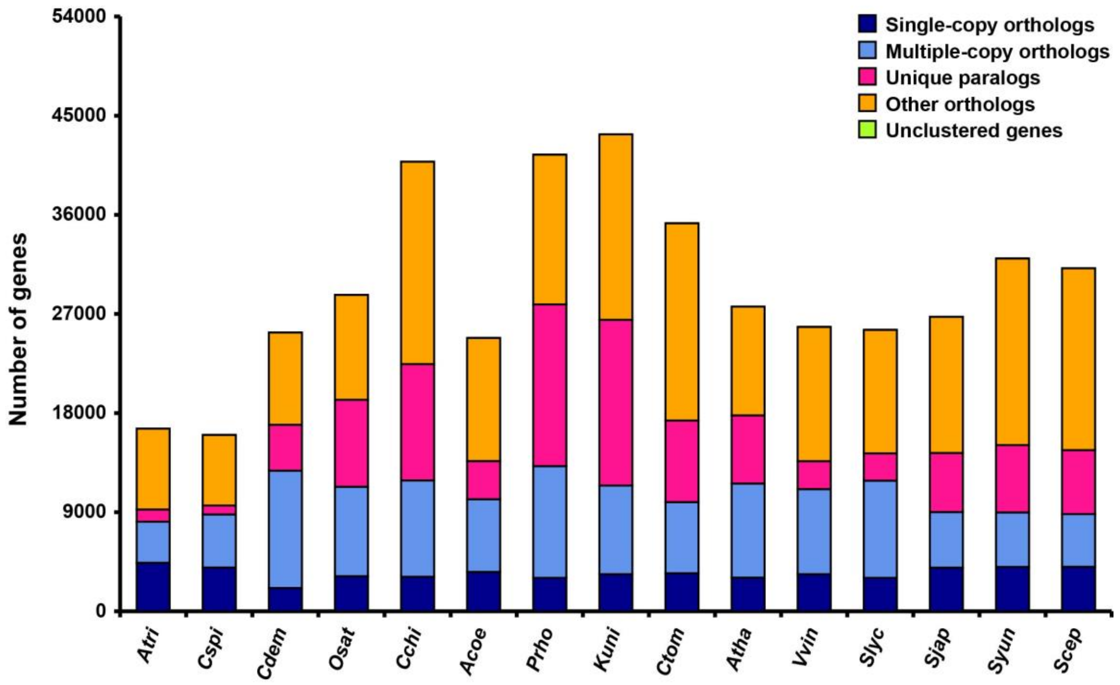

Supplementary Fig. 4. Number of ortholog gene family in 14 species.

Atri, *Amborella trichopoda*; Cdem, *Ceratophyllum demersum*; Osat, *Oryza sativa*; Cchi, *Coptis chinensis*; Acoe, *Aquilegia coerulea*; Prho, *Papaver rhoeas*; Kuni, *Kingdonia uniflora*; Ctom, *Corydalis tomentella*; Atha, *Arabidopsis thaliana*; Vvin, *Vitis vinifera*; Slyc, *Solanum lycopersicum*; Sjap, *Stephania japonica*; Syun, *Stephania yunnanensis*; Scep, *Stephania cepharantha*. Source data are provided as a Source Data file.

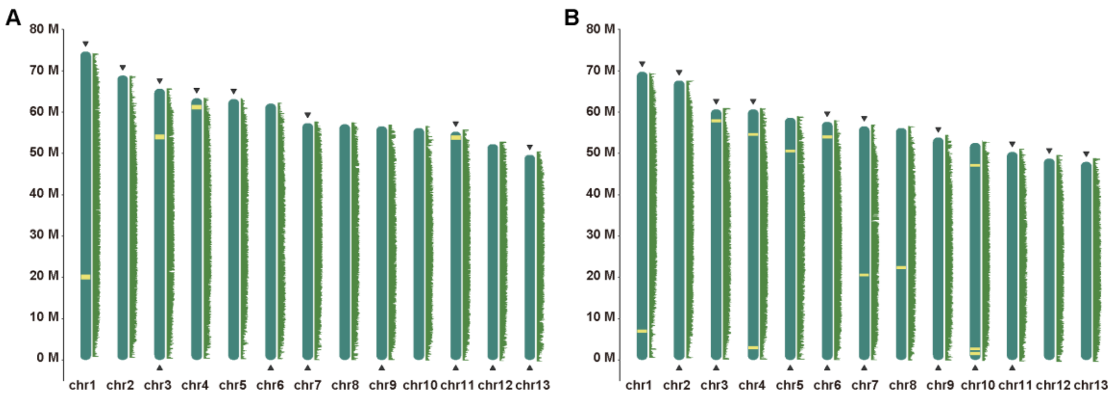

Supplementary Fig. 5. Illustration of the remaining gaps and annotated telomeres. The remaining gaps and annotated telomeres in *S. yunnanensis* (A) and *S. cepharantha* (B) were illustrated with yellow bars or black triangles. Yellow bars indicate remaining gaps after gap filling. Annotated telomere sequence repeats are shown as black triangles. Source data are provided as a Source Data file.

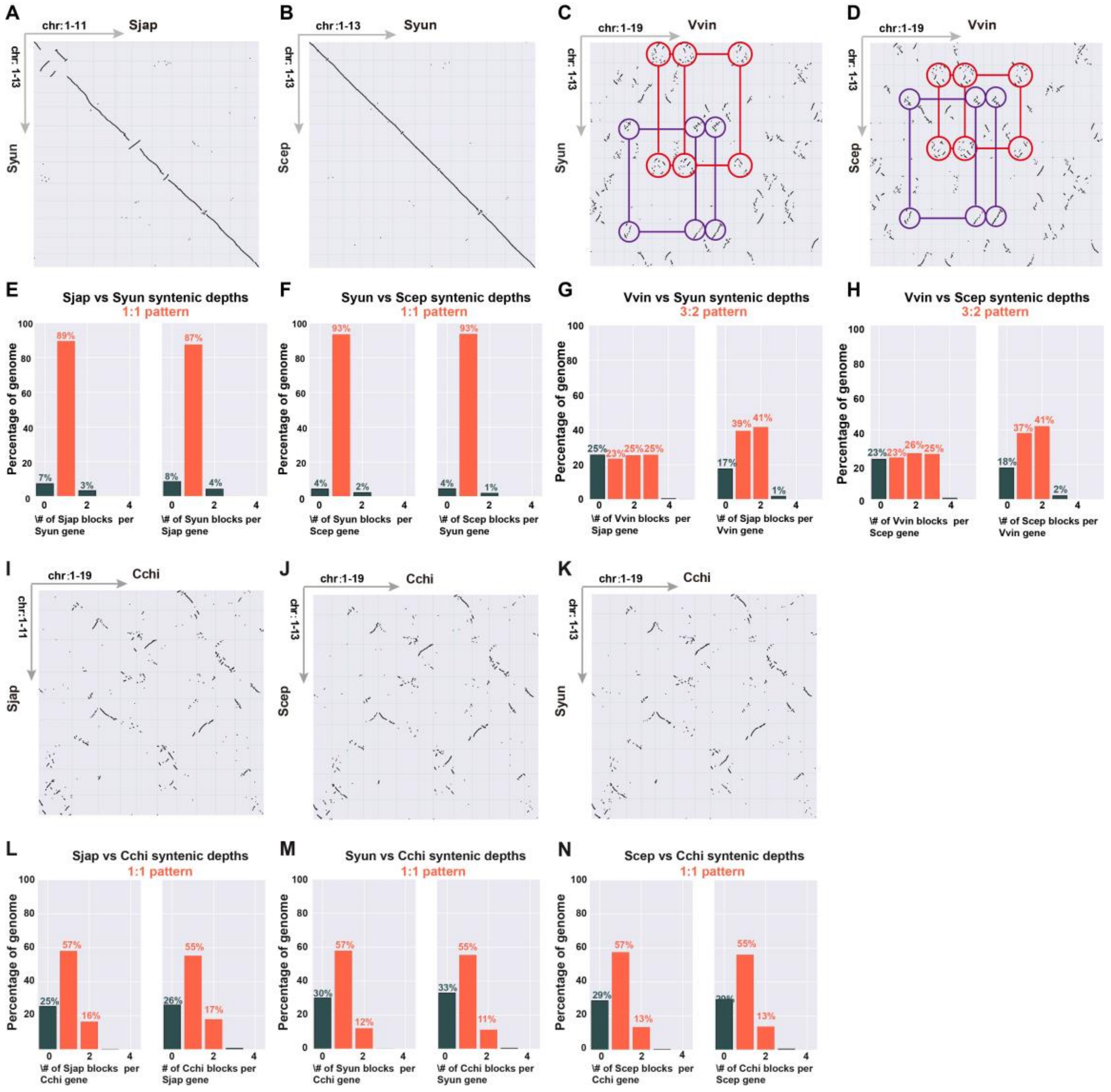

Supplementary Fig. 6. Synteny dot-plot and estimated synteny depths between three *Stephania* genomes and selected plant species.

Synteny dot-plots between *S. japonica*-*S. yunnanensis* (A), *S. japonica*-*S. cepharantha* (B), *V. vinifera*-*S. yunnanensis* (C), *V. vinifera*-*S. cepharantha* (D), *C. chinensis*-*S. japonica* (I), *C. chinensis*-*S. yunnanensis* (J), and *C. chinensis*-*S. cepharantha* (K). Synteny depths of *S. japonica* versus *S. yunnanensis* (E), and *S. cepharantha* (F) imply a 1:1 pattern. Synteny depths for *V. vinifera* with *S. yunnanensis* (G), and *S. cepharantha* (H) as 3:2 pattern, while synteny depths for *C. chinensis* with *S. japonica* (L), *S. yunnanensis* (M), and *S. cepharantha* (N) imply a 1:1 pattern. Source data are provided as a Source Data file.

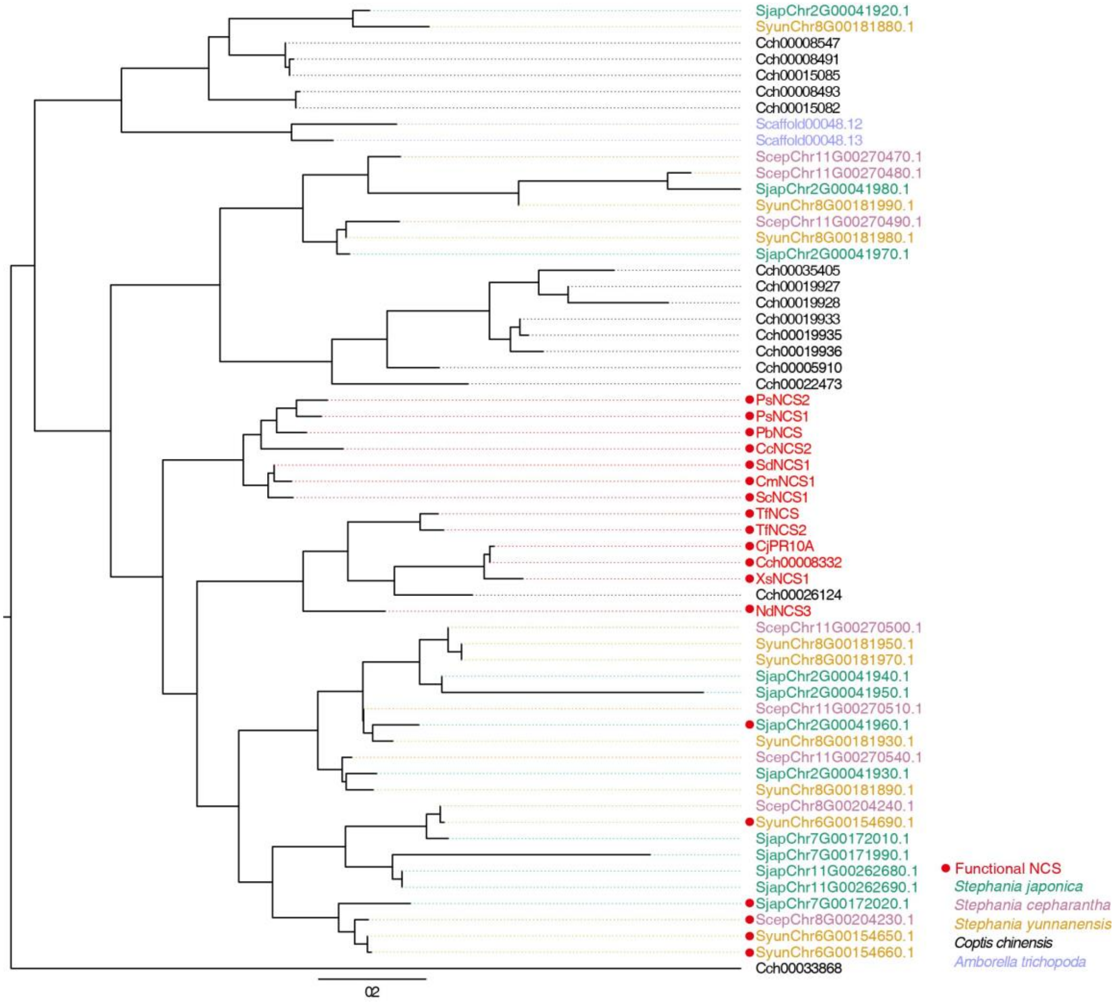

Supplementary Fig. 7. Phylogenetic relationship of *NCS* genes in different species.

Source data are provided as a Source Data file.

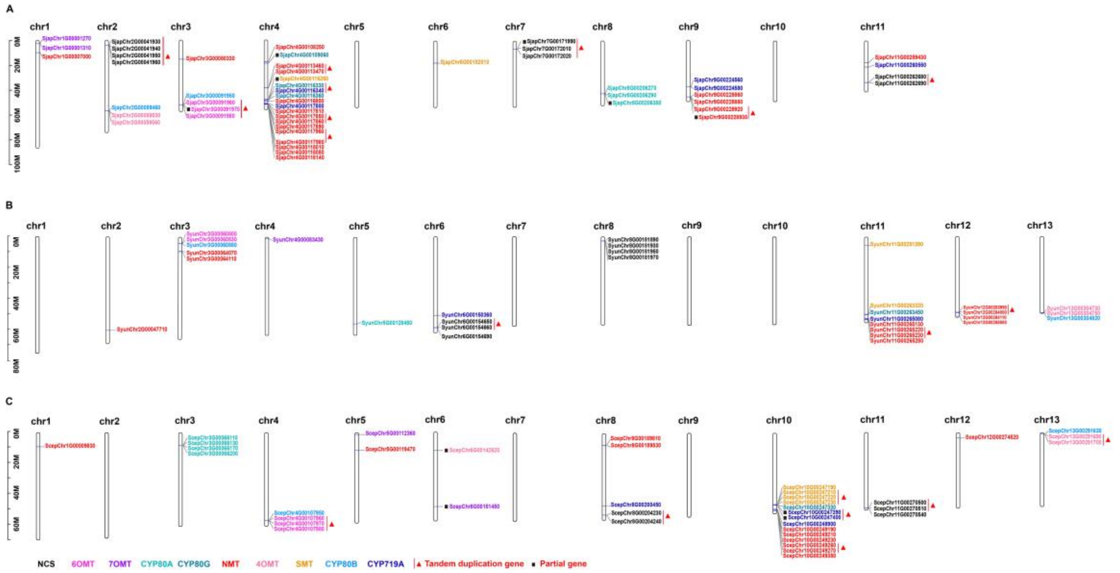

Supplementary Fig. 8. The chromosomal location of benzylisoquinoline alkaloids-biosynthesis genes in three *Stephania* species.

A, the chromosomal location of BIA-biosynthesis genes in *S. japonica*; B, the chromosomal location of BIA-biosynthesis genes in *S. yunnanensis*; C, the chromosomal location of BIA-biosynthesis genes in *S. cepharantha*. Source data are provided as a Source Data file.

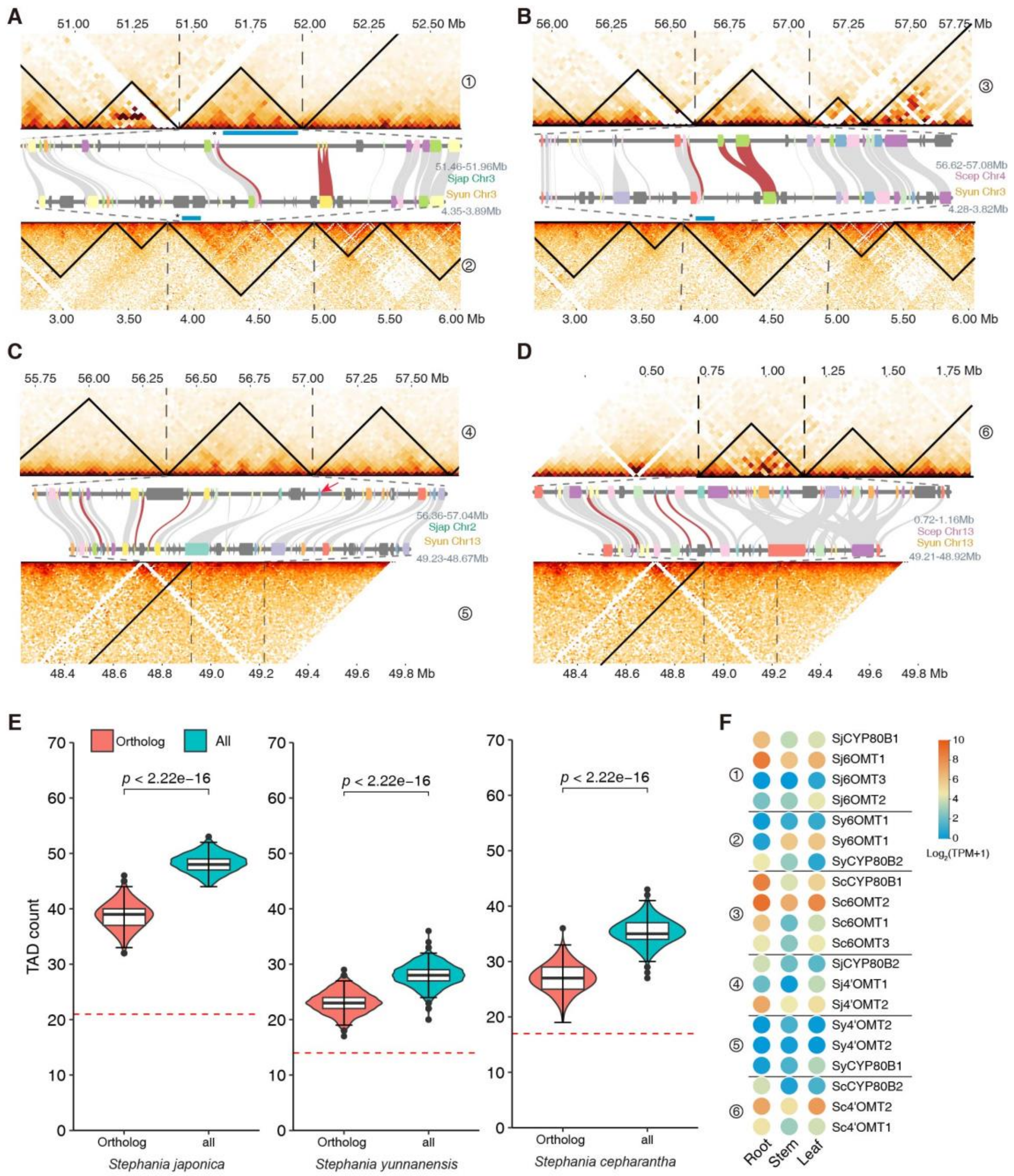

Supplementary Fig. 9. A putative biosynthetic gene cluster (BGC) located in three *Stephania* species.

(A-D) Illustration of a putative biosynthetic gene cluster (BGC) located in three *Stephania* species. The topologically associating domains (TADs) present in this cluster are conserved between these three species. (E) Expression of BGC genes. (F) Enrichment of candidate biosynthetic genes in the same TAD (box plot represents median and 25th and 75th percentiles—interquartile range; IQR—and whiskers extend to maximum and minimum values; n = 1000 permutations; statistical analysis: two-sided Wilcox test). Source data are provided as a Source Data file.

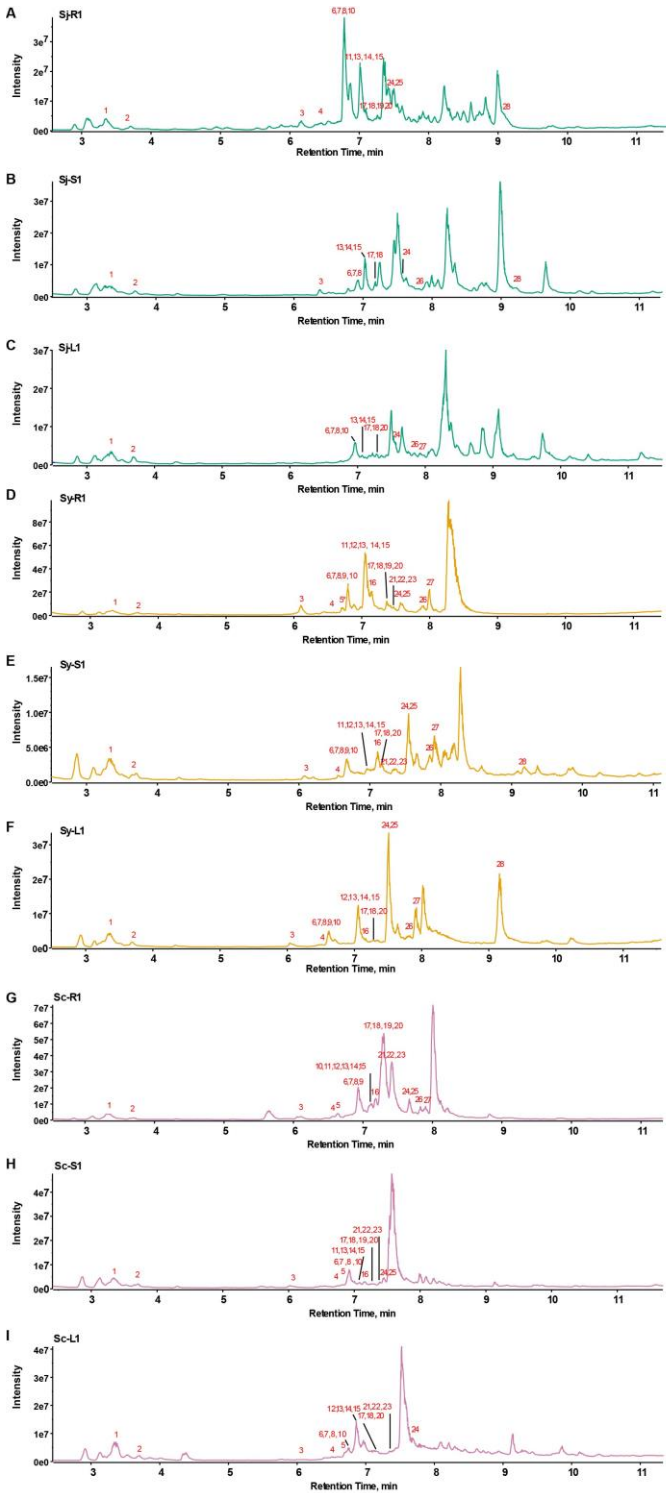

Supplementary Fig. 10. Determination of benzylisoquinoline alkaloids in different tissues of *Stephania* species by UPLC-MS.

A-C, Determination of benzylisoquinoline alkaloids in root, seed, and leaf of *S. japonica*; D-F, Determination of benzylisoquinoline alkaloids in root, seed, and leaf of *S. yunnanensis*; G-I, Determination of benzylisoquinoline alkaloids in root, seed, and leaf of *S. cepharantha*. Source data are provided as a Source Data file.

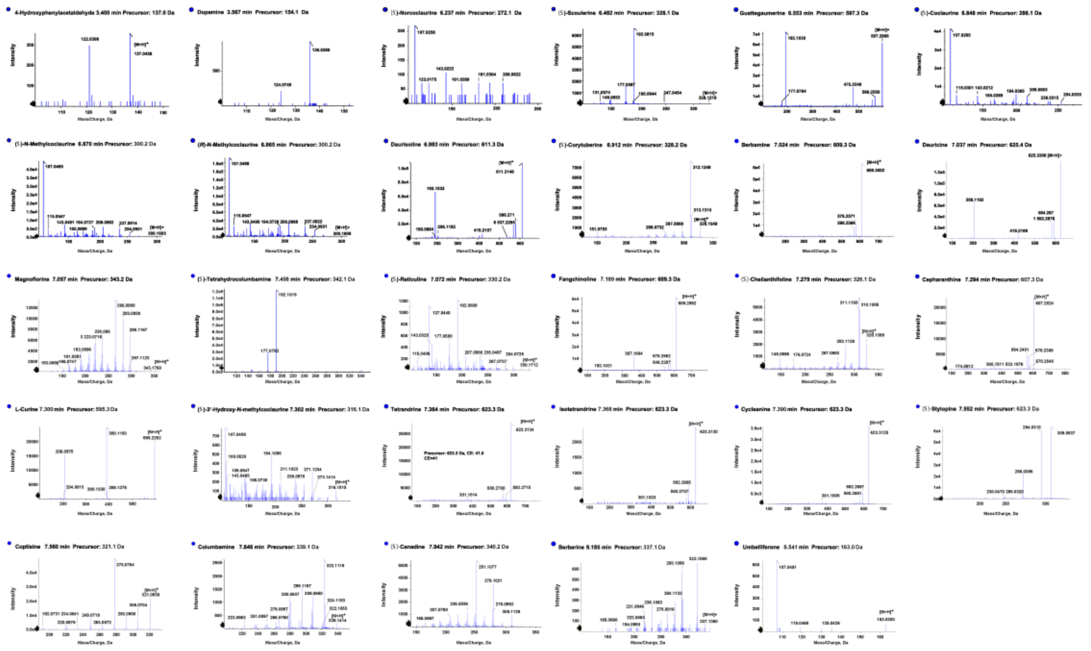

Supplementary Fig. 11. Mass spectrogram of 29 compounds by UPLC-MS/MS. Umbelliferone was as an internal standard. Source data are provided as a Source Data file.

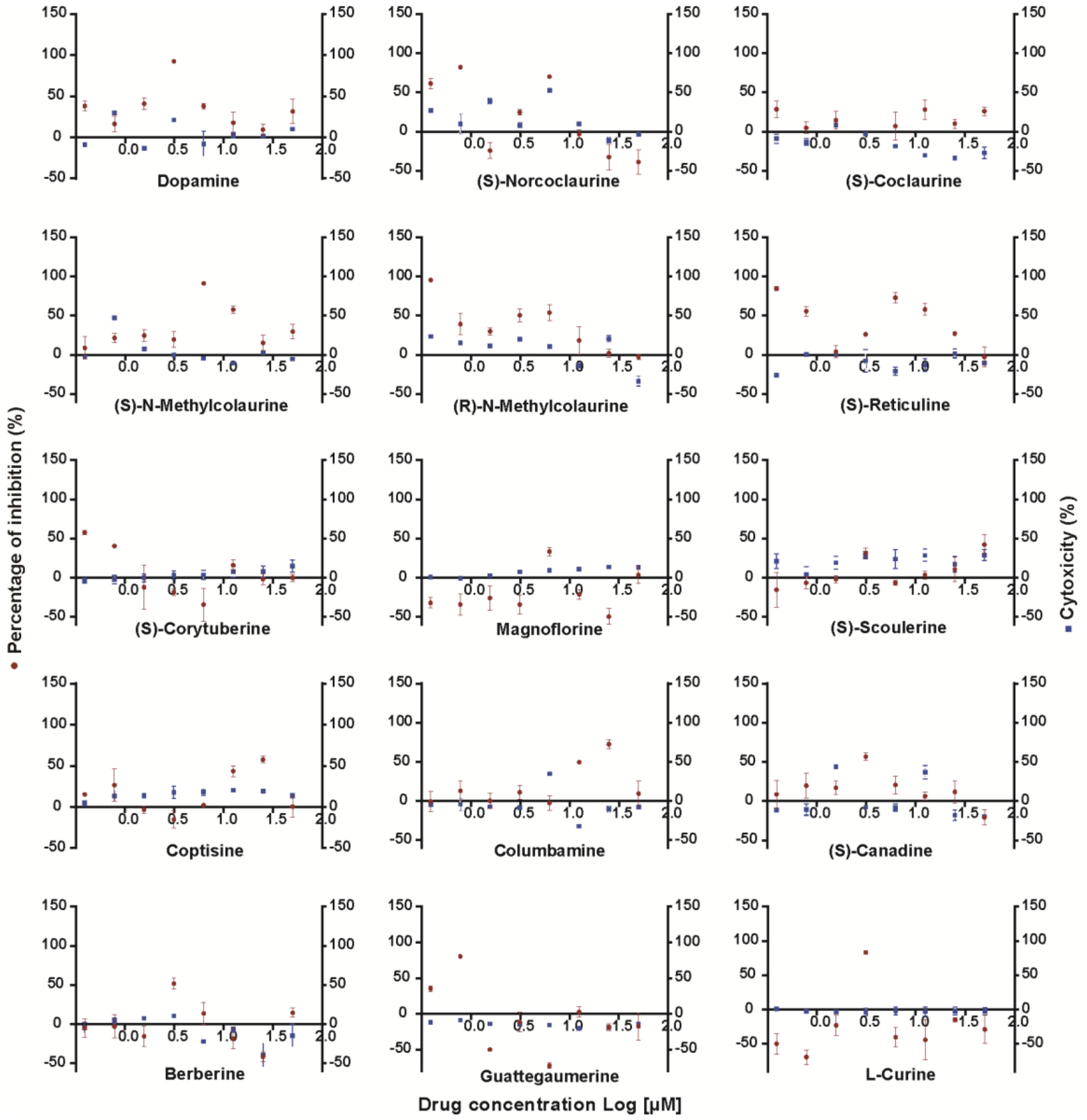

Supplementary Fig. 12. Anti-viral activities of 15 compounds against Guangxi pangolin-CoV (GX_P2V).

Dose-response were presented with red dots, while cytotoxicity percentages were denoted with blue squares. Source data are provided as a Source Data file.

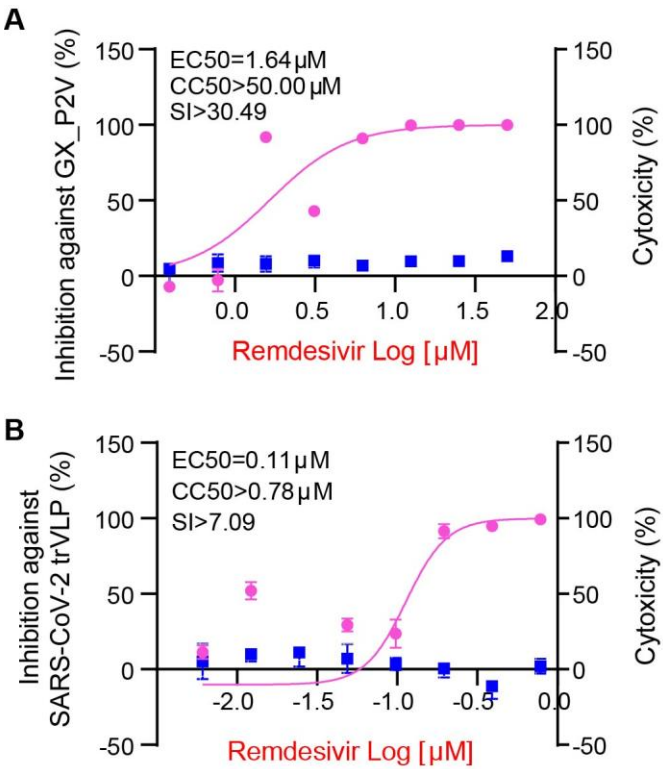

Supplementary Fig. 13. Anti-viral activities of remdesivir against Guangxi pangolin-CoV (GX_P2V) and SARS-CoV-2 trVLPs.

A, Anti-viral activities of remdesivir against GX_P2V; B, Anti-viral activities of remdesivir against SARS-CoV-2 trVLPs. Magenta points and lines denote the antiviral activity of the compounds against GX_P2V and SARS-CoV-2 trVLPs, respectively. The blue box represents cytotoxicity of Remedesivir. The data are presented as mean values +/- s.d. (n = 3 biologically independent samples). Source data are provided as a Source Data file.

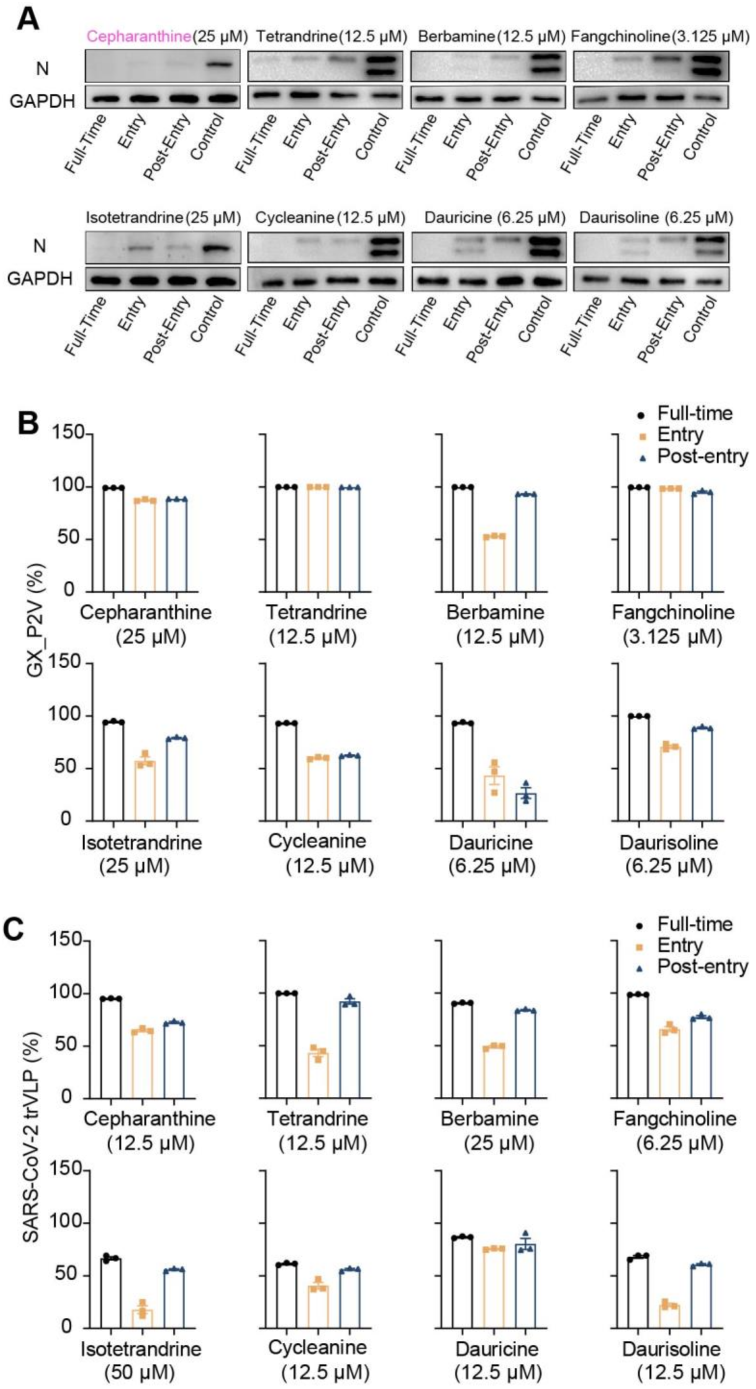

Supplementary Fig. 14. Antiviral activity assessment of eight BIAs.

(A) Western blot analysis of the nucleocapsid protein (NP) content in the time-of-addition assay of eight BIAs against GX_P2V. (B) and (C) Time-of-addition assay of eight BIAs against GX_P2V and SARS-CoV-2 trVLPs. Virus yield in the infected cell lysates was quantified by qRT-PCR. The data are presented as mean values +/- s.d. (n = 3 biologically independent samples). Source data are provided as a Source Data file.

Supplementary Table 1. Genomic features estimated by *k*-mer analysis.

Species	*k*-mer	*k*-mer number	*k*-mer depth	Estimated genome size (Mb)	Heterozygous ratio (%)	Duplication ratio (%)
S. japonica	19	85,239,681,549	133.90	636.6	0.93	56.69
S. yunnanensis	19	116,226,995,155	142.58	813.6	1.42	50.61
S. cepharantha	19	102,138,607,038	131.92	783.0	0.98	54.59

Supplementary Table 2. Summary statistics of ONT, DNBSEQ and Hi-C sequencing.

	S. japonica	*S. yunnanensis*	*S. cepharantha*
**Oxford Nanopore ultra-long sequencing**			
Total base (Gb)	97.46	81.24	88.25
Total reads	2,640,272	1,678,668	2,318,993
Maximum length (bp)	408,897	369,577	384,331
Average length (bp)	36,911.11	48,394.33	38,054.25
N50 (bp)	51,470	51,470	53,040
Depth (X)	151.48	100.02	118.70
**DNBSEQ sequencing**			
Total base (Gb)	118.19	158.71	124.99
Read length (bp)	150	150	150
Q20 (%)	97.79	98.37	98.08
Q30 (%)	92.61	93.95	93.57
Depth (X)	183.71	195.41	168.12
**Hi-C sequencing**			
Total base (Gb)	116.70	106.84	136.64
Read length (bp)	150	150	150
Q20 (%)	96.88%	98.46%	97.17%
Q30 (%)	90.34%	95.19%	91.26%
Depth (X)	181.39	131.55	183.79

Supplementary Table 3. The genome assembly statistics of *S. japonica*, *S. yunnanensis*, and *S. cepharantha*.

Assembly features	*S. japonica*	*S. yunnanensis*	*S. cepharantha*
Total length (bp)	643,414,607	812,193,072	742,977,745
N50 (bp)	22,324,849	37,605,797	6,848,000
L50	12	9	28
N90 (bp)	9,047,141	6,913,936	1,193,400
L90	29	31	132
Total length (bp)	643,369,795	812,198,542	743,462,617
N50 (bp)	54,031,414	56,656,017	50,698,408
L50	5	7	7
N90 (bp)	41,274,414	49,758,040	22,475,016
L90	10	13	14
Number of gaps before gap filling	52	47	229
Number of gaps after gap filling	1	4	11

Supplementary Table 4. Genome assembly quality assessment using BUSCO (embryophyta_odb10).

Species		BUSCO (embryophyta_odb10)
S. japonica	assembly	C:96.9% [S:95.0%, D:1.9%], F:0.9%, M:2.2%
	annotation	C:96.8% [S:94.6%, D:2.2%], F:0.9%, M:2.3%
S. yunnanensis	assembly	C:96.8% [S:93.8%, D:3.0%], F:0.9%, M:2.3%
	annotation	C:96.1% [S:93.2%, D:2.9%], F:1.3%, M:2.6%
S. cepharantha	assembly	C:96.0% [S:94.1%, D:1.9%], F:1.4%, M:2.6%
	annotation	C:96.2% [S:93.8%, D:2.4%], F:1.5%, M:2.3%

C, complete BUSCOs; S, complete single-copy BUSCOs; D, complete duplicated BUSCOs; F, fragmented BUSCOs; M, missing BUSCOs.

Supplementary Table 5. Summary of predicted genes.

	S. japonica	*S. yunnanensis*	*S. cepharantha*
Total number of gene	26,742	32,039	31,150
Average length of mRNA (bp)	6,423.78	6,724.15	6,297.33
Average length of CDS (bp)	1,068.27	961.77	938.24
Average exon number	5.46	5.12	4.94
Average length of exon (bp)	314.94	340.64	359.10
Average length of intron (bp)	1,048.10	1,203.42	1,143.97
Total number of exon	146,113	164,057	153,846
Total number of intron	119,371	132,018	122,696
Total intron length (bp)	125,113,321	158,872,573	140,360,662

Supplementary Table 6. Summary of repeat contents.

Type	*S. japonica*		*S. yunnanensis*		*S. cepharantha*	
	Length (bp)	Percent (%)	Length (bp)	Percent (%)	Length (bp)	Percent (%)
DNA	25,102,126	3.90	56,206,937	6.92	46,679,725	6.28
LINE	13,965,633	2.17	24,649,264	3.03	21,337,240	2.87
SINE	10,734	0.00	13,081	0.00	89,575	0.01
LTR	187,832,846	29.19	291,621,429	35.91	279,213,544	37.58
LTR-Gypsy	124,167,290	19.30	185,944,481	22.89	197,118,378	26.53
LTR-Copia	61,227,887	9.52	101,154,378	12.45	74,585,889	10.04
Satellite	563,268	0.09	363,435	0.04	224,615	0.03
Simple repeat	109,617	0.02	32,671	0.00	853,741	0.11
Other	3,452	0.00	8,668	0.00	7,620	0.00
Unknown	276,000,707	42.90	276,227,154	34.01	245,251,631	33.01
Total	477,412,695	74.20	626,599,430	77.15	573,339,339	77.17

Supplementary Table 7. The sequences of primers for candidate *NCS* gene searching.

Primer name	Sequences (5'-3')
ScepNCS1-F	gtcgcggatccgaattcATGATCAAGAAGGAGCTGAGACA
ScepNCS1-R	cgacggagctcgaattcCTAGAACATATCTGCGATAAATCTCG
ScepNCS2-F	gtcgcggatccgaattcATGATTAGGAAAGAACTCACACACG
ScepNCS2-R	cgacggagctcgaattcCTAATACTTGTATGCCATGACTCTTG
SjapNCS1-F	gtcgcggatccgaattcATGGAGAAGTACTGCATGATCAGG
SjapNCS1-R	cgacggagctcgaattcTTAATAAGGATTACCACGACCTAGAA
SjapNCS2-F	gtcgcggatccgaattcATGATCAGGAAGGAACTGAAACATG
SjapNCS2-R	cgacggagctcgaattcTCATGGCTTCTTCTTCTCTAAAAC
SjapNCS3-F	gtcgcggatccgaattcATGATTAGGAAAGAACTCACATACGA
SjapNCS3-R	cgacggagctcgaattcCTAATTCTTGTACGCCATGACTCTC
SjapNCS4-F	gtcgcggatccgaattcATGATCAAGAAGGAGCTCAAACAC
SjapNCS4-R	cgacggagctcgaattcCTAGAACATATCTGCGATAAATCTTG

Supplementary Table 8. List of compounds used for antiviral assays in this study.

Number	Compound	Molecular formula	Molecular weight	Company
1	Dopamine	$C_8H_{11}NO_2$	153.18	shchimie Bio-technology Co., Ltd.
2	(*S*)-norcoclaurine	$C_{16}H_{17}NO_3$	271.31	Chengdu Push Bio-technology Co., Ltd.
3	Coclaurine	$C_{17}H_{19}NO_3$	285.34	Sichuan Weikeqi Biological Technology CO., LTD
4	(*S*)-N-methylcoclaurine	$C_{18}H_{21}NO_3$	299.36	Toronto Research Chemicals-Canda
5	(*R*)-N-Methylcoclaurine	$C1_8H_{21}NO_3$	299.36	BioBioPha Co., Ltd.
6	(*S*)-reticuline	$C_{19}H_{23}NO_4$	329.39	BioBioPha Co., Ltd.
7	(*S*)-Scoulerine	$C_{19}H_{21}NO_4$	327.37	BioBioPha Co., Ltd.
8	Columbamine	$C_{20}H_{20}NO_4$	338.38	Shanghai yuanye Bio-Technology Co., Ltd
9	Berberine	$C_{20}H_{18}NO_4$	336.37	BioBioPha Co., Ltd.
10	(*S*)-corytuberine	$C_{19}H_{21}NO_4$	327.37	Chengdu Biopurify Phytochemicals Ltd.
11	Magnoflorine	$C_{20}H_{24}NO_4$	342.41	Chengdu Biopurify Phytochemicals Ltd.
12	Cepharanthine	$C_{37}H_{38}N_2O_6$	606.71	Chengdu Push Bio-technology Co., Ltd.
13	Tetrandrine	$C_{38}H4_2N_2O_6$	622.75	Chengdu Push Bio-technology Co., Ltd.
14	Berbamine	$C_{37}H_{40}N_2O_6$	608.72	Chengdu Biopurify Phytochemicals Ltd.
15	Fangchinoline	$C_{37}H_{40}N_2O_6$	608.72	Chengdu Biopurify Phytochemicals Ltd.
16	Isotetrandrine	$C_{38}H_{42}N_2O_6$	622.75	BioBioPha Co., Ltd.
17	Cycleanine	$C_{38}H_{42}N_2O_6$	622.75	Chengdu Herbpurify CO., LTD
18	L-curine	$C_{36}H_{38}N_2O_6$	594.7	Chengdu Herbpurify CO., LTD
19	Dauricine	$C_{38}H_{44}N_2O_6$	624.76	Chengdu Biopurify Phytochemicals Ltd.
20	Daurisoline	$C_{37}H_{42}N_2O_6$	610.75	Chengdu Biopurify Phytochemicals Ltd.
21	Dauriciline	$C_{36}H_{40}N_2O_6$	596.71	Chengdu Biopurify Phytochemicals Ltd.
22	Coptisine	$C_{19}H_{14}NO_4$	320.32	Shanghai yuanye Bio-Technology Co., Ltd
23	Thalidezine	$C_{38}H_{42}N_2O_7$	638.7	National Compound Library of Traditional Chinese Medicine
24	Liensinine	$C_{37}H_{42}N_2O_6$	610.7	National Compound Library of Traditional Chinese Medicine
25	Neferine	$C_{38}H_{44}N_2O_6$	624.8	National Compound Library of Traditional Chinese Medicine
26	Methoxyadiantifoline	$C_{43}H_{52}N_2O_{10}$	756.9	National Compound Library of Traditional Chinese Medicine
27	Thalmineline	$C_{42}H_{50}N_2O_{10}$	742.9	National Compound Library of Traditional Chinese Medicine

Supplementary Table 9. The sequences of primers for qRT-PCR experiments.

**Primer name**	**Sequences (5'-3')**
SARS-CoV-2 trVLP/GX_P2V-F	GGTGATTGCCTTGGTGATATTG
SARS-CoV-2 trVLP/GX_P2V-R	GCAAGTAGTGCAGAAGTGTATTG
PEDV-F	TCTGGTGGCTGCTGTCAA
PEDV-R	TGCTAAACTGGCGATCTG
SADS-CoV-F	GTTGATTGTAAGGCTTGGCG
SADS-CoV-R	AACCACACTTCCACTCAGC
GAPDH-F	AGCCTCAAGATCATCAGCAATG
GAPDH-R	ATGGACTGTGGTCATGAGTCCTT
