# Supplementary material for: Cepharanthine analogs mining and genomes of Stephania accelerate anti-coronavirus drug discovery
Source: Nat Commun. 2024 Feb 20;15:1537. doi: 10.1038/s41467-024-45690-5 (PMC10879537; doi:10.1038/s41467-024-45690-5)
Supplement: Supplementary file 3 — Description of Additional Supplementary Files [file 41467_2024_45690_MOESM3_ESM.pdf]

**Description of Additional Supplementary Files**

File Name: Supplementary Data 1
Description: The concentration of drugs against different coronavirus
