# Supplementary material for: Cepharanthine analogs mining and genomes of Stephania accelerate anti-coronavirus drug discovery
Source: Nat Commun. 2024 Feb 20;15:1537. doi: 10.1038/s41467-024-45690-5 (PMC10879537; doi:10.1038/s41467-024-45690-5)
Supplement: Supplementary file 5 — Reporting Summary [file 41467_2024_45690_MOESM5_ESM.pdf]

nature portfolio

Corresponding author(s): Huahao Fan, Chi Song, Yigang Tong, Shilin Chen

Last updated by author(s): Jan 21, 2024

Reporting Summary

Nature Portfolio wishes to improve the reproducibility of the work that we publish. This form provides structure for consistency and transparency in reporting. For further information on Nature Portfolio policies, see our Editorial Policies and the Editorial Policy Checklist.

Statistics

For all statistical analyses, confirm that the following items are present in the figure legend, table legend, main text, or Methods section.

n/a	Confirmed	
☐	☒	The exact sample size (*n*) for each experimental group/condition, given as a discrete number and unit of measurement
☐	☒	A statement on whether measurements were taken from distinct samples or whether the same sample was measured repeatedly
☐	☒	The statistical test(s) used AND whether they are one- or two-sided<br>*Only common tests should be described solely by name; describe more complex techniques in the Methods section.*
☒	☐	A description of all covariates tested
☒	☐	A description of any assumptions or corrections, such as tests of normality and adjustment for multiple comparisons
☐	☒	A full description of the statistical parameters including central tendency (e.g. means) or other basic estimates (e.g. regression coefficient) AND variation (e.g. standard deviation) or associated estimates of uncertainty (e.g. confidence intervals)
☐	☒	For null hypothesis testing, the test statistic (e.g. *F*, *t*, *r*) with confidence intervals, effect sizes, degrees of freedom and *P* value noted<br>*Give P values as exact values whenever suitable.*
☒	☐	For Bayesian analysis, information on the choice of priors and Markov chain Monte Carlo settings
☒	☐	For hierarchical and complex designs, identification of the appropriate level for tests and full reporting of outcomes
☒	☐	Estimates of effect sizes (e.g. Cohen's *d*, Pearson's *r*), indicating how they were calculated

Our web collection on statistics for biologists contains articles on many of the points above.

Software and code

Policy information about availability of computer code

Data collection	Applied Biosystems was used to process qRT-PCR data.
Data analysis	We used public softwares including SOAPnuke v2.1.4, GUPPY v0.3.0, GCEpackage v1.0.0, NextDenovo v2.5.0, Racon v1.4.11, Pilon v1.23, ALLHIC v0.9.12, Juicebox V1.11.08, Winnowmap v1.11, Purge haplotigs pipeline v.1.0.4, medaka consensu v1.2.1, nucmer v3.1, bedtools v2.30.0, RepeatModeler v.1.0.4, RepeatMasker v.4.0.5, tRNAscan-SE v1.23, INFERNAL v1.1.2, Minimap2 v2.17, stringtie2 v2.1.5, TransDecoder v5.1.0, Augustus v3.3.2, Genscan v1.0, GlimmerHMM v3.0.4, Exonerate v2.4.0, MAKER v2.31.10, BUSCO v5.2.2, BLASTP v.2.6.0, interProScan v5.33, HMMER v3.1, KOBAS v3.0, diamond v2.0.4.142, Orthofinder v.2.3.12, RAxML v8.2.10, MCMCtree v4.9, MCScanX, HiC-Pro v3.1.0, HiCExplorer v3.7.2, plantiSMASH23 v1.0, CAFÉ v3.1, TBtools V1.098761, AutoDockVina v1.2.5, AutoDockTools v1.5.7, and Graph PadPrism 8.

For manuscripts utilizing custom algorithms or software that are central to the research but not yet described in published literature, software must be made available to editors and reviewers. We strongly encourage code deposition in a community repository (e.g. GitHub). See the Nature Portfolio guidelines for submitting code & software for further information.

Data

Policy information about availability of data

All manuscripts must include a data availability statement. This statement should provide the following information, where applicable:

- Accession codes, unique identifiers, or web links for publicly available datasets
- A description of any restrictions on data availability
- For clinical datasets or third party data, please ensure that the statement adheres to our policy

The three Stephania genome assemblies generated by this study have been archived under the China National GeneBank DataBase (CNGBdb) accession number CNP0003595 (https://db.cngb.org/search/?q=CNP0003595). All raw sequencing data were deposited under the National Center for Biotechnology Information (NCBI) GenBank accession number PRJNA888087 (https://dataview.ncbi.nlm.nih.gov/object/PRJNA888087?reviewer=1m7fajgt91c9k1ceh8n8qia5go). Source data in this study are provided in figshare (https://doi.org/10.6084/m9.figshare.23887953). The information of functional BIAs-biosynthetic genes and compounds are hosted in GitHub (https://github.com/liuzy2008/evo-chemo_anti-SARS-CoV-2_drug_discovery2023).
Source data are provided in this paper.

Research involving human participants, their data, or biological material

Policy information about studies with human participants or human data. See also policy information about sex, gender (identity/presentation), and sexual orientation and race, ethnicity and racism.

Reporting on sex and gender	This study did not involve human participants.
Reporting on race, ethnicity, or other socially relevant groupings	This study did not involve human participants.
Population characteristics	This study did not involve human participants.
Recruitment	This study did not involve human participants.
Ethics oversight	No ethical approval or guidance was required since we did not perform relevant experiments.

Note that full information on the approval of the study protocol must also be provided in the manuscript.

Field-specific reporting

Please select the one below that is the best fit for your research. If you are not sure, read the appropriate sections before making your selection.

[x] Life sciences [ ] Behavioural & social sciences [ ] Ecological, evolutionary & environmental sciences

For a reference copy of the document with all sections, see nature.com/documents/nr-reporting-summary-flat.pdf

Life sciences study design

All studies must disclose on these points even when the disclosure is negative.

Sample size	To investigate the content of BIAs in different tissues of the three Stephania species, a ultra-performance liquid chromatography–tandem mass spectrometry system (SCIEX TripleTOF 6600+), ultra-high–performance liquid chromatography (ultra-HPLC) coupled with time-of-flight mass spectrometry was used for the relative quantification of metabolites. We have finally identified and tested the antiviral activity of 28 compounds.
Data exclusions	No data is excluded.
Replication	The antiviral assays were performed in biological triplicates. For metabolome analysis, three biological replicates were performed. All attempts at replication were successful.
Randomization	For RNA-Seq and HPLC analysis, the samples were allocated into experimental groups at random.
Blinding	Blinding is not applicable in our study because it does not involve subjects which receive different treatments. All experiments were done by analyzing data derived from experiment or sequenced data directly.

Reporting for specific materials, systems and methods

We require information from authors about some types of materials, experimental systems and methods used in many studies. Here, indicate whether each material, system or method listed is relevant to your study. If you are not sure if a list item applies to your research, read the appropriate section before selecting a response.

Materials & experimental systems

n/a	Involved in the study	
☐	☒	Antibodies
☐	☒	Eukaryotic cell lines
☒	☐	Palaeontology and archaeology
☒	☐	Animals and other organisms
☒	☐	Clinical data
☒	☐	Dual use research of concern
☐	☒	Plants

Methods

n/a	Involved in the study	
☒	☐	ChIP-seq
☒	☐	Flow cytometry
☒	☐	MRI-based neuroimaging

Antibodies

Antibodies used	SARS-CoV-2 Nucleocapsid Antibody (3F9), mAb, Mouse (Genscript, catalog No. A02049100); GAPDH Monoclonal antibody (Proteintech, catalog No. 60004-1-Ig); HRP-conjugated Affinipure Goat Anti-Mouse IgG (H+L) (Proteintech, catalog No.SA00001-1).
Validation	All of the antibodies used in this study were purchased from company. SARS-CoV-2 Nucleocapsid Antibody (3F9) was purchased from Genscript (catalog No. A02049100); GAPDH Monoclonal antibody was purchased from Proteintech (catalog No. 60004-1-Ig); HRP-conjugated Affinipure Goat Anti-Mouse IgG (H+L) was purchased from Proteintech (catalog No.SA00001-1).

Eukaryotic cell lines

Policy information about cell lines and Sex and Gender in Research

Cell line source(s)	Vero E6 cells, Huh7 cells, Caco2-N, BHK21-ACE2.
Authentication	Vero E6 and Huh-7 cells were Purchased from NICR (National Infrastructure of Cell Line Resource); Caco2-N cells were kindly provided by Professor Ding Qing of Tsinghua University; BHK21-ACE2 cells were kindly provided by Xu Huan in ShenZhen Bar Laboratories. None of the cell lines used in this study were found in the list of known misidentified cell lines.
Mycoplasma contamination	The cells were regularly detected for mycoplasma contamination using the MycAwayTM Plus-Color one-step Mycoplasma Detection kit (Yysen Biotechnology, China), and all the used cells were negative for mycoplasma contamination during use period.
Commonly misidentified lines (See ICLAC register)	None.
